# Functional heterogeneity of meniscal fibrochondrocytes and microtissue models is dependent on modality of fibrochondrocyte isolation

**DOI:** 10.1111/cpr.13735

**Published:** 2024-10-08

**Authors:** Zhiyao Ma, Shikha Chawla, Xiaoyi Lan, Eva Zhou, Aillette Mulet‐Sierra, Melanie Kunze, Mark Sommerfeldt, Adetola B. Adesida

**Affiliations:** ^1^ Department of Surgery, Faculty of Medicine and Dentistry University of Alberta Edmonton Alberta Canada; ^2^ Department of Biomedical Engineering, Faculty of Engineering University of Alberta Edmonton Alberta Canada

## Abstract

Collagenase digestion (d) and cellular outgrowth (og) are the current modalities of meniscus fibrochondrocytes (MFC) isolation for bioengineering and mechanobiology‐related studies. However, the impact of these modalities on study outcomes is unknown. Here, we show that og‐ and d‐isolated MFC have distinct proliferative capacities, transcriptomic profiles via RNA sequencing (RNAseq), extracellular matrix (ECM)‐forming, and migratory capacities. Our data indicate that microtissue pellet models developed from og‐isolated MFC display a contractile phenotype with higher expressions of alpha‐smooth muscle actin (*ACTA2*) and transgelin (*TAGLN*) and are mechanically stiffer than their counterparts from d‐MFC. Moreover, we introduce a novel method of MFC isolation designated digestion‐after‐outgrowth (dog). The transcriptomic profile of dog‐MFC is distinct from d‐ and og‐MFC, including a higher expression of mechanosensing caveolae‐associated caveolin‐1 (*CAV1*). Additionally, dog‐MFC were superior chondrogenically and generated larger‐size microtissue pellet models containing a higher frequency of smaller collagen fibre diameters. Thus, we demonstrate that the modalities of MFC isolation influence the downstream outcomes of bioengineering and mechanobiology‐related studies.

## Graphical abstract

Functional heterogeneity of human meniscal fibrochondrocytes is dependent on modality of isolation.

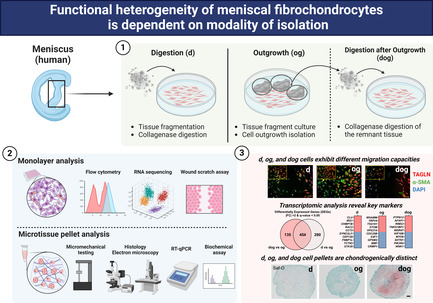

## 1 INTRODUCTION

Meniscus is the vital fibrocartilaginous tissue of the knee joint composed primarily of water 72% and 28% of collagen fibre and other extracellular matrix (ECM) components like glycosaminoglycans.^1^ Meniscus performs the essential function of shielding the articular cartilage tissue from load and shocks, making itself vulnerable to injuries.^2^ The inner avascular region of the meniscus shows similarity with articular cartilage, containing rounded and polygonal cells that display high gene expression of collagen type II and aggrecan, while the outer vascular region of meniscus demonstrates high gene expression of collagen type I with cells showing fibroblast‐like cells.^3^, ^4^

Since the peripheral outer meniscus is vascularized, and the inner meniscus is not, it is probable that avascularity contributes to compromised healing of the inner meniscus.^4^ Ultimately, the loss of meniscus tissue, whether from traumatic tears, repetitive injuries, or age‐related degeneration, subjects the articular cartilage lining the tibial plateau and femoral condyles to higher peak stresses, which inadvertently leads to degradation and osteoarthritis (OA).^5^ Therefore, it is imperative to make concerted efforts to engineer meniscus to minimise the chances for OA development.

Cell‐based strategies have been used in the past to promote meniscus regeneration. Numpaisal et al. used partially digested articular chondrocytes for the regeneration of meniscus tissue.^6^ Hatsushika et al. reported intra‐articular injections of allogeneic synovial mesenchymal stem cells (MSC) appeared to promote meniscus regeneration and provide protection to the medial femoral articular cartilage in a porcine massive meniscal defect model.^7^ Marsano et al. compared different cell sources, including articular chondrocytes, synovial membrane cells, human inner meniscus cells and fat pad cells, for developing meniscus substitutes. They showed that only articular chondrocytes generated tissues with significant glycosaminoglycan (GAG) deposition and cell phenotypes like the inner and outer meniscus regions.^8^ Nevertheless, from a clinical perspective, the ideal tissue for the isolation of meniscus fibrochondrocytes (MFC) for meniscus regeneration is the meniscus undergoing meniscectomy because no further morbidity is generated. Furthermore, as highlighted in our previous study, cells obtained from native meniscus are inherently primed to synthesise the functional ECM of meniscus and are reported to have superior fibrochondrogenic differentiation potential compared to MSC.^9^

Research for meniscus regeneration using MFC has progressively moved towards clinical phases. Several studies have mentioned the use of wide variety of enzymatic digestion methods in which proteolytic enzymes are used to obtain single‐cell suspension from tissue.^3^, ^10^, ^11^ A few reported the use of explant culture method where no enzyme is used; native tissue is cut into smaller pieces, which are placed in culture dishes, and cells then start to migrate out of tissue and adhere to the culture surface for the isolation of MFC from human meniscus tissue. In the case of the explant culture, the native tissue remains active during the process of primary culture and can release growth factors into the media, and migrated cells may continue their communication with the native tissue and modulate secretory events supporting the migrating cells.^12^ Nevertheless, there has been little close focus on the selection of precise method to be used for cell isolation, evaluating phenotypic and genotypic properties specific to their use in meniscus repair and regeneration. The isolation methods of MFC are not always consistent among researchers, and no study has investigated the impact of isolation methods on cellular phenotype and mechanobiology outcomes. It is important to highlight that a critical part unexploited until now is what happens to the cells in the remnant tissue after it is deprived of the outgrowth cells. Therefore, in the current study, we were motivated to isolate and study the cells in the remnant tissue for their meniscus regeneration properties and thus, for the first time, report a third novel method of cell isolation where the remnant tissue after outgrowth (og) cells was taken through enzymatic digestion to isolate the remaining cells called the digestion‐after‐outgrowth (dog) cells.

The current study aimed to deduce the differences in the phenotype of cell populations isolated using different isolation methods: digestion (d), outgrowth (og), and digestion after outgrowth (dog). Further, using the microtissue pellet model, we evaluated how these different cell isolation methods affect morphology and chondrogenic differentiation capacities as measured by ECM synthesis, expression of chondrogenic markers, microtissue mechanical properties, expression of contractile markers alpha‐smooth muscle actin (*ACTA2*) and Transgelin (*TAGLN*), and migration of the cells. Additionally, we conducted a detailed transcriptomics analysis of monolayer cells cultured under hypoxia (3% O_2_) before the formation of microtissue pellets, aiming to explore potential links between the molecular profiles of the differentially isolated cells and the characteristics of their corresponding microtissue pellets.

## 2 RESULTS AND DISCUSSION

### 2.1 Surface signatures of cells isolated via d, og and dog methods

**FIGURE 1:**
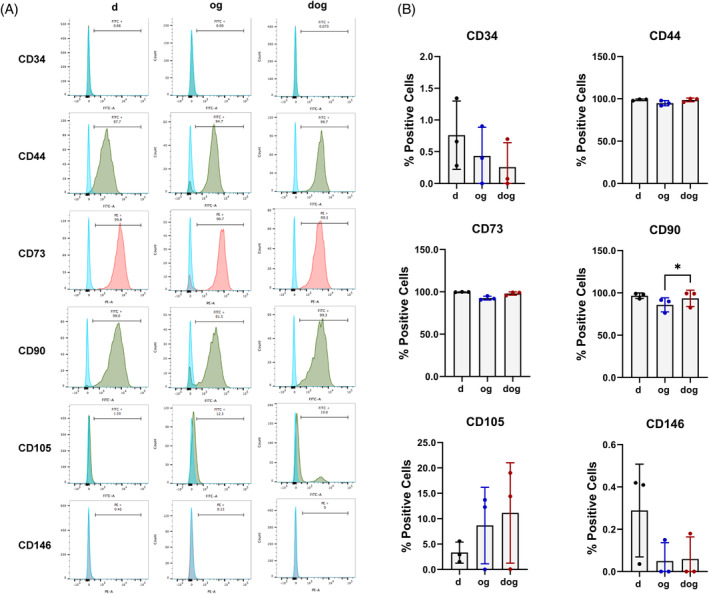
Cell Surface Marker Profiling of Differentially Isolated Meniscus Cells via Flow Cytometry. (A) Representative histograms depict the expression profiles of selected surface markers (CD34, CD44, CD73, CD90, CD105, and CD146) in comparison to isotype controls. (B) Quantification of the percentage of cells expressing each selected marker (*n* = 3).

We selected a panel of cell surface markers that had been used to characterise human meniscus cells using FACS flow cytometry.^13^ Additionally, we assessed the expression of the cell surface marker, CD146, which is expressed on perivascular cells,^14^ as a potential discriminator between cells from the vascular and avascular regions of the meniscus. With flow cytometry, we demonstrated that regardless of the modality of cell isolation, the cells were >95% positive for CD44, CD73, and CD90 (Figure 1). CD44 serves as the hyaluronan receptor and has been shown to be expressed on the surface of meniscus cells,^13^ and widely expressed on the surface of most cell types, including chondrocytes with high chondrogenic capacities^15^ and bone marrow mesenchymal stem cells (BMSC).^16^ CD73 is a cell‐surface ecto‐enzyme (ecto‐5′‐nucleotidase) that catalyses the dephosphorylation of extracellular AMP into adenosine, which in turn activates the G protein–coupled receptors to exert potent immunoregulatory activity and is primarily expressed on the surface of immune regulatory T cells.^17^ CD73 is also expressed on the surface of human BMSC^16^ and chondrogenic subpopulations of synovium‐derived mesenchymal stem cells.^18^ CD90 or Thy‐1 is a glycophosphatidylinositol (GPI) cell surface protein that is highly expressed on the surface of mesenchymal stem cells^19^ and articular chondrocytes.^20^ The high expression levels of CD90 in all groups of the differentially isolated human MFC confirmed their mesenchymal origin (Figure 1A,B). The significantly lower expression of CD90 in the og‐method isolated MFC relative to the dog‐method isolated MFC suggests that the chondrogenic capacity of the dog‐method isolated cells is superior.^15^ CD105, or endoglin, is expressed on the surface of endothelial cells^21^ and MSC.^16^ The expression of CD105 on MFC was dependent on the method of cell isolation (Figure 1A,B). For two of the three donors, the MFC isolated via the og and dog methods were CD105 positive and those via the d method, regardless of the donor, were almost negative. In contrast, the cells were almost negative for CD34 and CD146. CD34, or sialomucin, is expressed in early haematopoietic and vascular‐associated tissue^22^ and in a minority population of human meniscus cells of the superficial zone with little to no matrix‐forming capacity.^13^, ^23^ CD146 also known as the melanoma cell adhesion molecule (MCAM) or cell surface glycoprotein MUC18^21^ is expressed in the majority of cell types, including endothelial, pericytes, smooth muscle cell, epithelia, fibroblasts, mesenchymal stem cells, and lymphocytes, with the exception of erythrocytes.^21^, ^24^ Collectively, the lack of CD34 and CD146, and the expression of other markers suggests that the cells in this study originated from the avascular portion of the meniscus.

### 2.2 Cells isolated via d, og and dog methods exhibit distinct chondrogenic capacities

**FIGURE 2:**
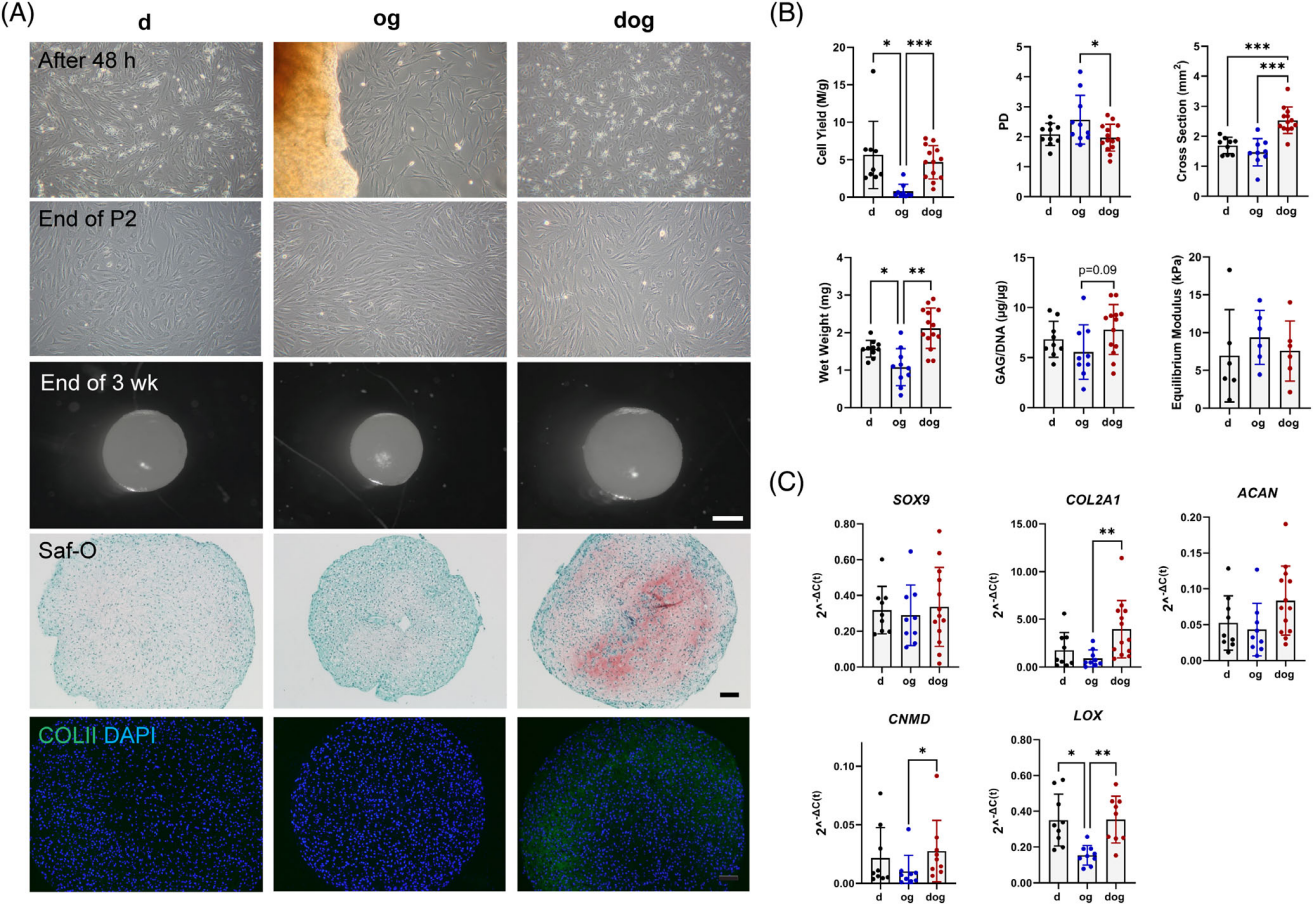
Comparative Analysis of Chondrogenic Potential in Microtissue Pellets Derived from d, og, and dog Isolation Methods. (A) Morphological comparison of meniscus cells isolated via d, og, and dog methods after a 48‐h recovery period and at the end of passage 2 (P2). Microtissue pellet morphology after a three‐week culture period. Histological and immunofluorescent visualisation for glycosaminoglycan (GAG) synthesis (Safranin O staining) and type II collagen deposition. Scale bars: White: 500 μm, Black: 100 μm. (B) Quantitative analysis of cell yield and population doubling at the end of P2. Comparison of microtissue pellet size, wet weight, GAG/DNA content, and mechanical properties following a three‐week culture period. (C) RT‐qPCR expression profile of selected chondrogenic markers (*SOX9, COL2A1, CNMD, ACAN,* and *LOX*) across the three groups. *p < 0.05; **p < 0.005; ***p < 0.001.

To identify potential distinctions between the cells obtained via the three isolation methods, we compared the features of cells obtained through d, og, and dog methods, along with an evaluation of the characteristics of the resulting microtissue pellets. The morphology of cells isolated by the d and dog methods exhibited similarity after 48 h of recovery period and at the conclusion of P2, depicting a mixed population of polygonal and spindle‐shaped cells (Figure 2A). This finding was consistent with the morphological description of collagenase‐mediated isolated human meniscus cells by Nakata et al.^11^ In the case of the og‐derived cells, the cells exhibited predominantly a fibroblast‐like, spindle‐shaped morphology with a mixture of cells oriented both parallelly and 180° to the diced meniscus explants after 48 h (Figure 2A). At the end of P2, the appearance of og cells was predominantly elongated and spindle‐shaped, fibroblast‐like (Figure 2A). Moreover, the cumulative population doubling of the og‐derived cells was significantly higher at the end of P2 compared to dog cells, with approximately a 10‐fold significantly lower cell yield compared to d and dog methods (Figure 2B).

Microtissue pellets were formed from 0.25 million P2 cells for all three isolation methods. After three weeks of in vitro chondrogenic microtissue pellet culture, microtissue pellets developed from the og method of cell isolation were the smallest in size and wet weight, while dog cells‐derived microtissue pellets exhibited the largest size and wet weight. Specifically, the cross‐sectional area of the microtissue pellets from dog cells was significantly 1.7 times larger than that of the pellets derived from og cells, and 1.5 times larger than those derived from d cells. Regarding wet weight, the microtissue pellets from dog cells were also significantly 2 times heavier than those derived from og cells (Figure 2B). Beyond the differences in size and wet weights, the microtissue pellets from different isolation methods also demonstrated distinct chondrogenic gene expression. The expression levels of selected chondrogenic markers (*SOX9, COL2A1, CNMD,* and *ACAN*) followed a similar trend as the size and weight, with dog pellets displaying the highest average expression levels of these chondrogenic markers, and og‐derived microtissue pellets had the least expression (Figure 2C). Due to large donor variability, only the comparison between og and dog group was statistically significant for *COL2A1*. Furthermore, *LOX*, the gene encoding lysyl oxidase—a collagen cross‐linking enzyme, was least expressed in og cells‐derived microtissue pellets and more expressed in dog and d cells‐derived microtissue pellets. The chondrogenic capacity, as determined by the value of the GAG/DNA ratio, was highest in microtissue pellets from dog isolated cells and, in contrast, was least in microtissue pellets from og cells (Figure 2B). This outcome of distinct chondrogenic capacities was further supported by the notably intense Safranin O staining and type II collagen immunofluorescence in dog‐derived microtissue sections (Figure 2A). In contrast, the equilibrium modulus of the microtissue pellets was highest in the og‐derived microtissue pellets (Figure 2B). Due to large donor variability, these outcomes were not statistically different.

### 2.3 Microtissue pellets differ in cytoskeletal and ECM structures

Next, we sought to explore the potential mechanisms underlying the different phenotypes of the microtissue pellets developed from the d, og, and dog methods. Given the migratory or cellular outgrowth nature of cell populations isolated by og method, and the contracted phenotype of their microtissue pellets, we directed our attention to examining the cytoskeletal and ECM features of all the microtissue pellets, regardless of the method of cell isolation.

**FIGURE 3:**
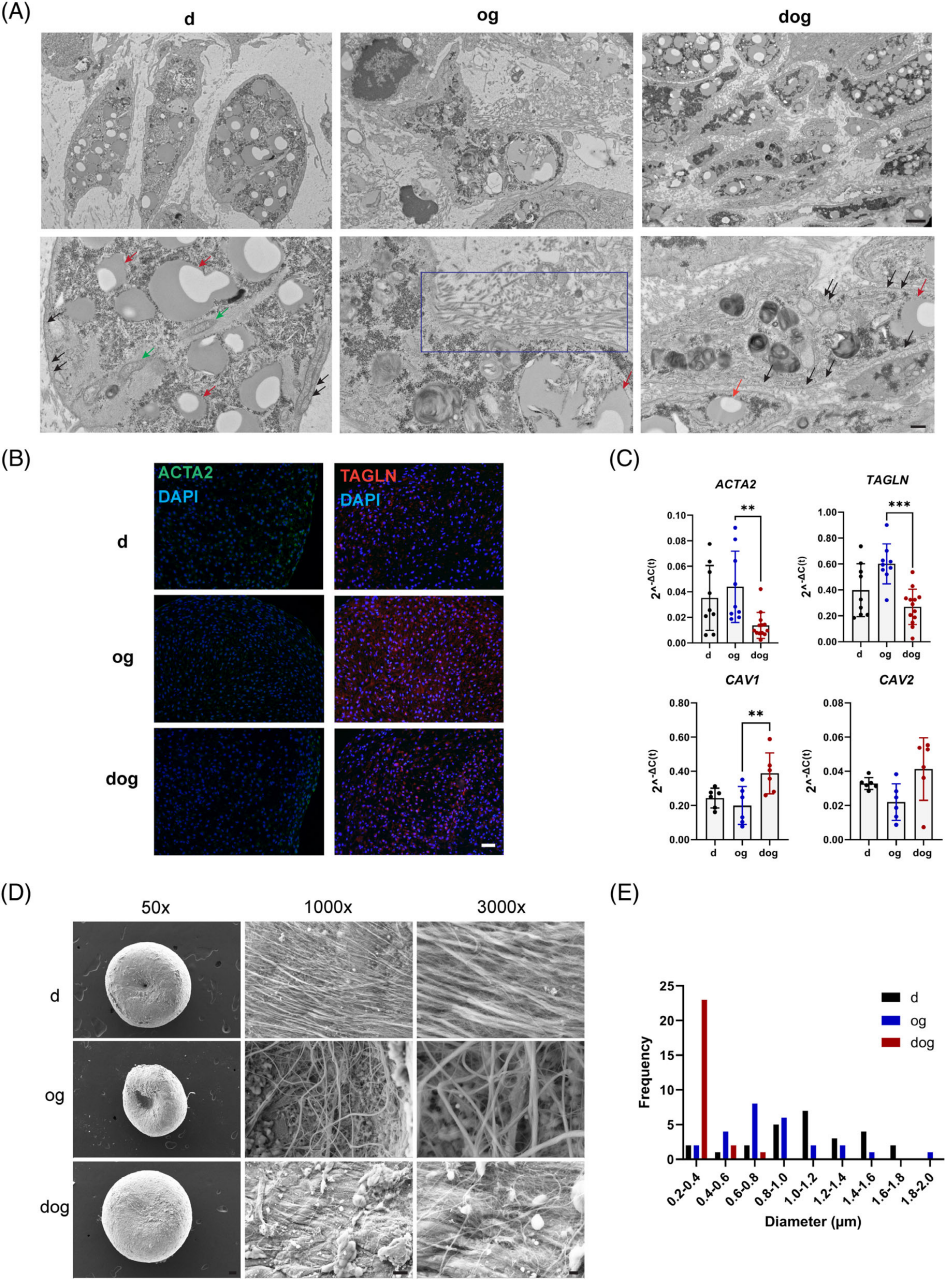
Ultrastructural Examination of Cytoskeleton and ECM Features in Microtissue Pellets from d, og and dog Isolation Methods. (A) Transmission electron microscopy (TEM) images showcase the ultrastructure of microtissue pellets, highlighting lipid droplets (red arrow), mitochondria (green arrow), caveolae (black arrow), and pericellular collagen fibres (blue box). Scale bars: Top panel: 2 μm, Bottom panel: 500 nm. (B) Immunofluorescent visualisation of transgelin (TAGLN) and alpha‐smooth muscle Actin (ACTA2) in the microtissue pellets. Scale bar: 50 μm (C) RT‐qPCR assessment of alpha‐smooth muscle Actin (*ACTA2*), transgelin (*TAGLN*), caveolin 1 (*CAV1*), and caveolin 2 (*CAV2*). (D) Scanning electron microscopy (SEM) images depict the collagen fibre network in the ECM fibre Scale bars: 50x: 100 μm, 1000x: 10 μm, 3000x: 2 μm. (E) Quantification of fibre diameter within the ECM. **p < 0.005; ***p < 0.001.

The ultrastructural features of the microtissue pellets were examined using transmission electron microscopy (TEM) (Figure 3A). A prominent feature observed for all cells from the three cell isolation methods was the presence of lipid droplets, appearing as uniform circular structures within the cells. Notably, cells isolated through the d and dog method exhibited comparable lipid droplets, while og cells showed the least amount. Additionally, a substantial number of mitochondria were exclusively observed in d cells, surrounded by abundant endoplasmic reticulum (Figure 3A). Collectively, the observed lipid droplets and the presence of mitochondria suggest potential differences in the bioenergetics of the differently isolated cells. The og‐isolated cells exhibited the most abundant deposition of pericellular collagen fibres. These fibres were in proximity to the cell membrane and extended into the pericellular space in a predominantly unidirectional orientation. In contrast, the collagen fibres were less abundant and organised around cells isolated by the dog method. The cells isolated via the d method seemed to lack these features (Figure 3A).

Another noteworthy structure observed was the mechanosensitive lipid‐rich plasma membrane invaginations named caveolae (Figure 3A).^25^, ^26^, ^27^ These mechanosensitive membrane invaginations, approximately 60–80 nm in diameter, were abundant in cells isolated from the dog method. While some were present in the d cells, none were found on the membrane of og cells. This observation was further supported by the expression levels of caveolin 1 and 2 (*CAV1*, and *CAV2*), which are two of three structural proteins of caveolae, with caveolin 3 o(*CAV3*) being the third but highly expressed in muscles.^26^ For *CAV1*, the dog‐derived microtissue pellets showed a significantly higher expression level than in the og microtissues, while the expression level in the d cells‐derived microtissue pellets was in between. The expression levels of caveolin 2 (*CAV2*) followed the same trend as *CAV1* but at ~10‐fold less the expression level of *CAV1* (Figure 3C).^27^ Collectively, this finding suggests the possibility that the response of d‐, og‐ and dog‐isolated meniscus cells to mechanical loading may be different. This possibility is in part supported by Szojka et al., where dynamic compression of human meniscus models developed from d‐isolated MFC significantly upregulated *CAV1*.^28^

In addition to the observed migratory characteristics of the og‐isolated cells, the corresponding microtissue pellets were significantly smaller in wet weight and cross‐sectional area, indicating tissue contraction, relative to microtissue pellets developed from d and dog isolated cells (Figure 2B). Moreover, the expression levels of two cytoskeletal protein encoding genes: *ACTA2* and *TAGLN*, which have been implicated in the acquisition of the contractile phenotype of dedifferentiated articular chondrocytes through actin polymerisation and myocardin‐related transcription factor‐a (MRTF‐A) nuclear localisation mechanisms, were highest in the microtissues developed from og‐isolated meniscus cells (Figure 3B,C).^29^

The collagen fibre network within the microtissue pellets' ECM was visualised and quantified using SEM (Figure 3D,E). Fibres present in both d and og pellets displayed a spectrum of diameters, spanning from 0.2 to 2 μm, with the d group peaking at 1.2 μm and the og group at 0.8 μm. Conversely, the dog group exhibited a more uniformly distributed fibre diameter at a relatively smaller size ~0.4 μm compared to the d and og groups. In addition, for the dog group, underneath the more uniformly distributed collagen fibre diameters appears to be distinctly wide lamellar bundles of collagen fibres as previously described beneath the superficial layer's collagen network of the human meniscus.^30^ Concerning the organisation of the fibre network, both d and dog groups exhibited a unidirectional orientation, whereas the fibres in the og group displayed a more randomly oriented configuration. The lack of preferred orientation of fibres in the og group is consistent with the arrangement of collagen fibres in the superficial layer of the femoral surface of the human meniscus. In contrast, a more organised fibre network is observed in the central layer of the human meniscus.^30^

### 2.4 Transcriptomic signatures underlie cellular and microtissue level differences

To decipher potential links between the molecular profiles of the differentially isolated cells and the characteristics of their corresponding microtissue pellets, we performed bulk RNAseq on monolayer cultured d‐, og‐ and dog‐isolated MFC at the end of P2. This enabled us to relate early‐stage molecular markers that could serve as predictive indicators for the characteristic features of the corresponding microtissue.

**FIGURE 4:**
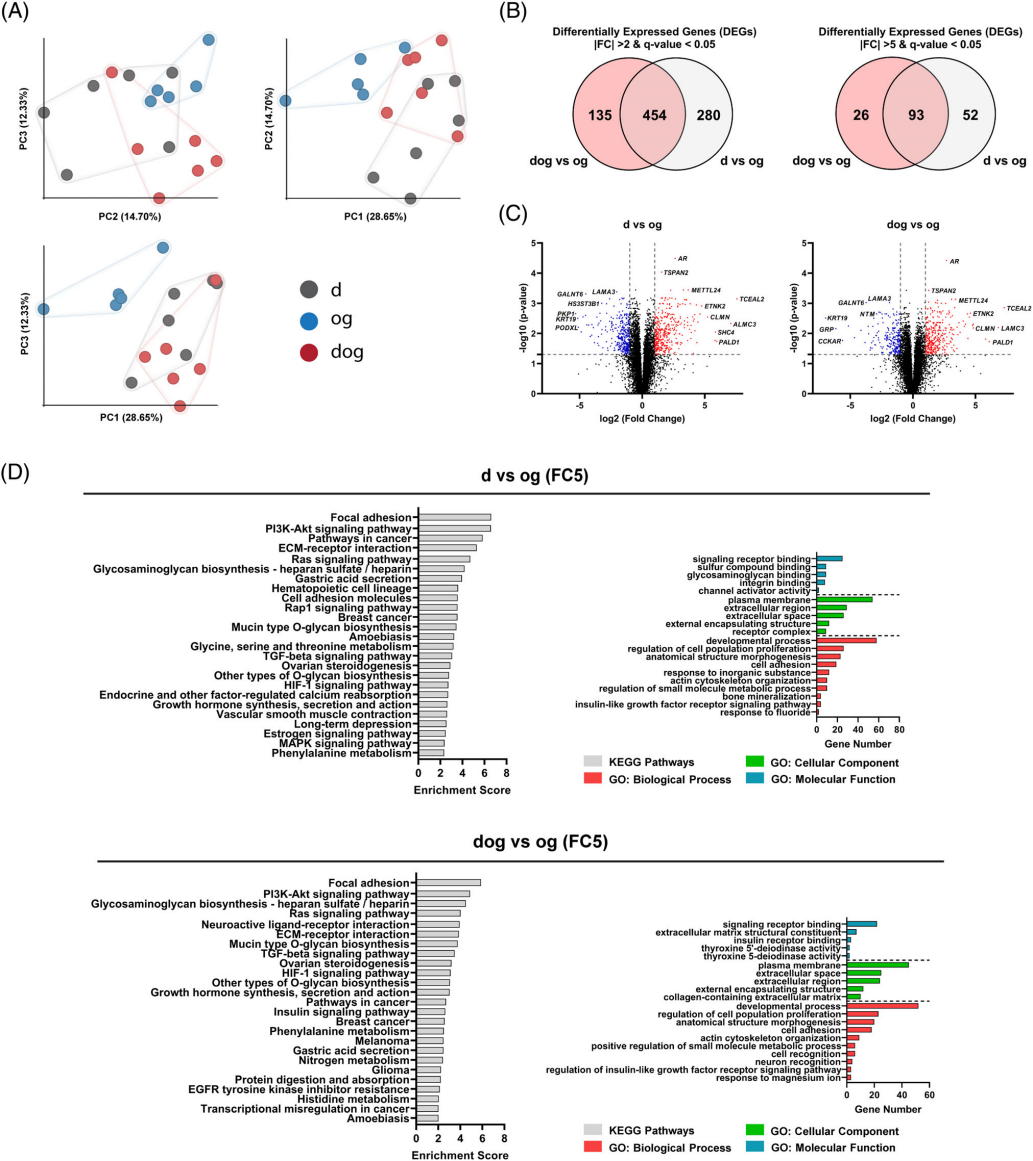
Transcriptomic Analysis and Functional Enrichment of Differentially Isolated Monolayer MFC. (A) PCA plots of transcriptomic profiles from d‐, og‐ and dog‐isolated meniscus cells show notable separation between og‐isolated cells and the others, on principal components 1 (PC1) and 3 (PC3). (B) Venn diagram showing the overlap of DEGs between d‐ vs. og‐ and dog‐ vs. og‐isolated cells with absolute fold changes (FC) of 2 and 5. (C) Volcano plot of DEGs (FC2), with significantly upregulated genes in red and downregulated genes in blue. (D) Functional enrichment analysis for DEGs with an FC of 5, highlighting top Gene Ontology (GO) terms and Kyoto Encyclopedia of Genes and Genomes (KEGG) pathways.

The principal component analysis (PCA) plots of the transcriptomic profiles of the differentially isolated cells revealed notable separations (Figure 4A). Of these separated plots, the PCA plot of the og‐isolated cells was the most distinct population, implying considerable transcriptomic profile differences relative to those of d‐ and dog‐isolated MFC population This is particularly evident on the principal component 1 (PC1) and PC3 plot (Figure 4A). To further explore the distinct transcriptomic profile of the og‐isolated cells, the transcriptomic expression of og‐isolated cells was employed as the denominator for subsequent analyses.

Upon pairwise comparison of the Differentially Expressed Genes (DEGs) between d‐ and og‐isolated cells or between dog‐ and og‐isolated cells, the majority of DEGs were found to be shared by two groups. The Venn diagram illustrated 454 genes shared between the pairwise comparisons with an absolute fold change (FC) of 2, and 93 genes with a FC of 5. However, there remained a small number of genes unique to each pairwise comparison, highlighting distinct differences between the d‐ and dog‐isolated MFC populations (Figure 4B). This trend was similarly observed in the volcano plot, where the two pairwise comparisons shared a panel of DEGs, with a small proportion of different genes (Figure 4C).

To enhance our understanding of the biological implications underlying the observed differences, we performed functional enrichment analyses based on the identified DEGs with both FC of 2 (Supplementary Figure S1) and 5 (Figure 4D). We focused on the FC of 5 for its direct relevance to cartilage‐related activities, providing a more focused view relative to the broader terms associated with an FC of 2. The top enriched non‐redundant Gene Ontology (GO) terms highlighted commonalities between pairwise comparisons of d‐ versus og‐, and dog‐ versus og‐, particularly in cellular components related to the ECM and plasma membrane. Notably, a unique cellular component in d vs og was identified as the ‘*receptor complex*’. Shared biological processes included terms such as ‘*cell adhesion*’, ‘*actin cytoskeleton organization*’ and ‘*regulation of cell population proliferation*’. These shared terms suggest consistent cellular behaviour while indicating the distinct profile of og‐isolated MFC regarding cytoskeletal structure and cell proliferation, the latter of which was supported by the highest population doublings (PD) of the og‐isolated MFC (Figure 2B). The most notable differences between the two pairwise comparisons were observed in molecular functions. The majority of top‐enriched Kyoto Encylopedia of Genes and Genomes (KEGG) pathways were common between comparisons, featuring cartilage‐related activities like ‘*glycosaminoglycan biosynthesis‐heparan sulfate/heparin*’, ‘*TGF‐beta signaling pathways*’, and ‘*PI3K‐Akt signaling pathway*’. The most significant enrichments were observed in pathways related to the interplay between the cytoskeleton and ECM, specifically ‘*focal adhesion*’ and ‘*ECM‐receptor interaction*’, further highlighting the distinct characteristics of og‐isolated MFC in cytoskeletal structure and contractile phenotype.

**FIGURE 5:**
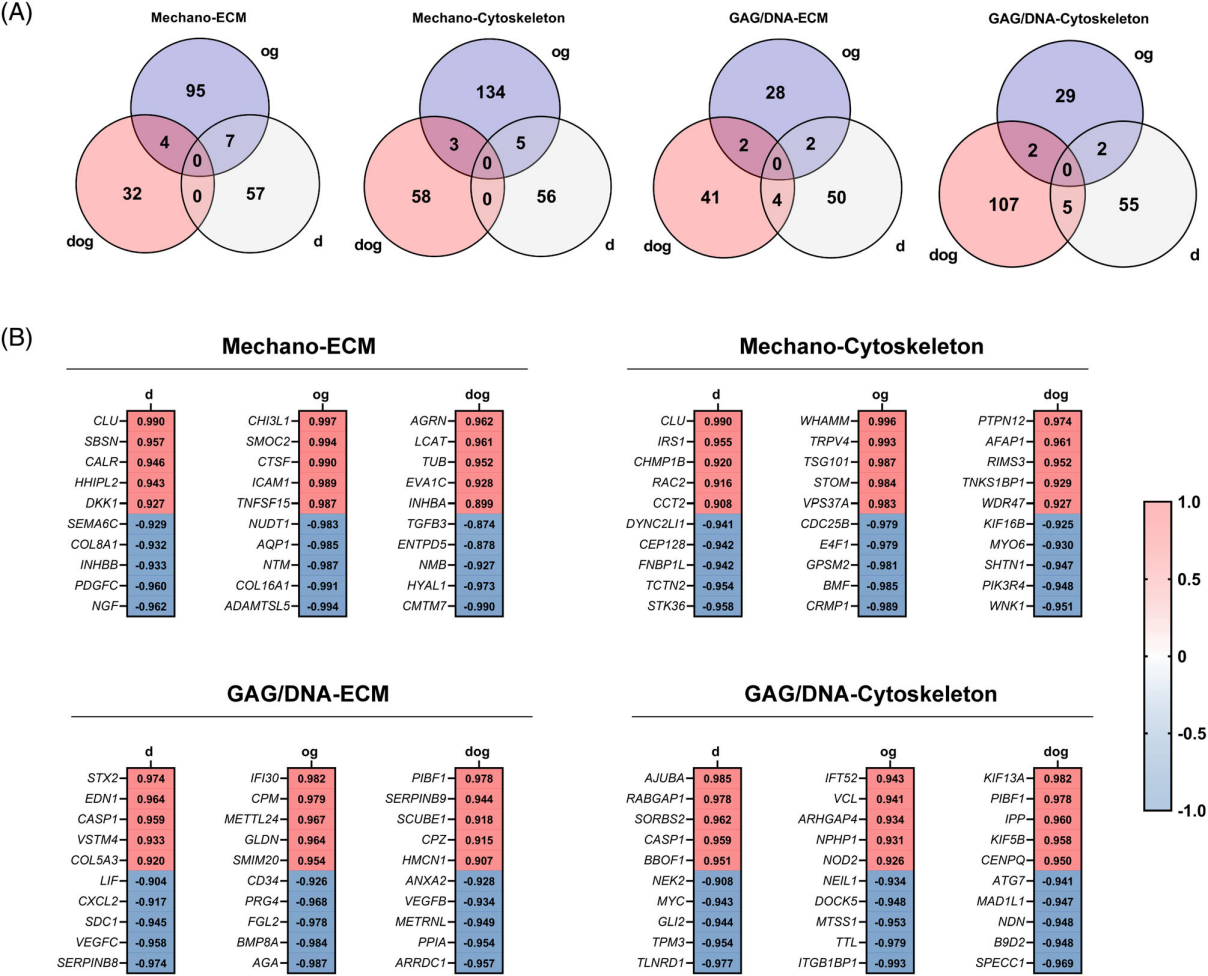
Correlation Analysis Between Monolayer Transcriptomic Profiles and Microtissue Characteristics. (A) Venn diagrams of genes from d‐, og‐ and dog‐isolated MFC with significant correlations to the biomechanical (equilibrium modulus) and biochemical (GAG/DNA content) properties of the corresponding microtissue pellets. (B) Heatmap showing the top five genes with the strongest positive or negative correlation coefficients for each category. Values indicate Pearson correlation coefficients.

To determine the genes expressed by monolayer MFC that may predict the biomechanical and biochemical characteristics of the microtissue pellets, we performed a correlation analysis between transcriptomic profiles of the d‐, og‐ and dog‐isolated MFC and the corresponding biomechanical (equilibrium modulus) and biochemical (GAG) characteristics at the microtissue pellet level, Subsequently, we screened the genes with significant positive or negative correlations (Figure 5A), and identified the top five genes with the strongest correlation coefficients from each category (Figure 5B). The identified genes with significant correlations were unique to d‐, og‐ and dog‐ groups, indicating distinctive gene signatures associated with the biomechanical and biochemical properties of the resultant microtissue pellets. These top‐correlated genes hold potential as predictors of the functional outcomes of microtissue pellets depending on the MFC isolation method.

The positively correlated genes in the d‐isolated MFC in terms of microtissue mechanical property (mechano‐) and cytoskeletal correlations included *CCT2*, which has been implicated in increased proliferation and migration of squamous carcinoma cells,^31^ and *CLU*, which has been identified to regulate cell proliferation and migration by activating protein kinase B (Akt)‐ pathway.^32^ Moreover, there was a positive correlation of *RAC2*, a member of Rac‐type GTPases reported to be involved in reorganising actin in migrating mammalian cells and enhancing nuclear yes‐associated protein (YAP)/transcriptional coactivator with PDZ‐binding motif (TAZ).^33^ *IRS1* was also positively correlated. *IRS1* has been reported to enhance the expression of TAZ and downstream signalling that modulates cell proliferation and migration.^34^ The negatively correlated cytoskeletal genes with microtissue mechanical property included a list of genes involved in ciliogenesis, including *STK36*, required for motile ciliogenesis^35^; *TCTN2*, depletion resulted in loss of ciliary membrane proteins and cilium shortening^36^; *CEP128*, that has been reported to regulate ciliary signalling^37^; and *DYNC2LI1*, that plays a significant role primary cilia formation in fibroblasts.^38^ Cilia are conserved microtubular elements that contribute to cell motility.^39^ Zhao et al. highlighted the significance of primary cilium in regulating mechanotransduction pathways in chondrocytes, the presence of primary cilia in meniscus was demonstrated by Graverand et al. in rabbit meniscus cells. They reported that primary cilia in meniscus functions as part of load sensory systems that are present within the meniscus similar to primary cilia identified in articular cartilage where cilia is required for mechanotransduction and associated ECM synthesis.^40^, ^41^

In the case of the Mechano‐cytoskeleton gene correlations of og‐isolated MFC, *VPS37A* was among the top five positively correlated genes. (Figure 5B). *VPS37A* plays a role in curvature sensing, an important phenomenon adapted by the migrating cells to navigate through tissues.^42^ Furthermore, *STOM*, which plays a role in mechanotransduction^43^; *TSG101*, which plays a role in remodelling of actin filaments^44^; *TRPV4*, which plays a role in mechanotransduction in chondrocytes^45^, ^46^ and ECM collagen remodelling^47^, ^48^ in gingival fibroblasts; and *WHAMM*, a downstream Rho signalling element that plays a role in regulating cytoskeletal dynamics and enhanced cell migration,^49^ were also positively correlated. As for the negatively correlated genes, *CRMP1*, which has been implicated in inhibiting prostate metastasis^50^; *GPSM2*, which is essential in maintaining cell polarity and cell cycle regulation^51^; *E4F1*, which is essential for cell cycle progression in stem cells; and *CDC25B*, a human phosphatase that are involved in cell‐cycle progression and division,^52^, ^53^ all significantly negatively correlated with the mechanical properties of microtissue pellets made by og‐isolated MFC. Another important observation for the og‐isolated MFC is the positive correlation between the expression of Tumour Necrosis Factor Ligand Superfamily Member 15 (*TNFSF15*) and the mechanical properties of the resultant microtissue pellets (Figure 5B). *TNFSF15*, a member of the TNF superfamily, is associated with the senescence‐associated secretory phenotype (SASP), which can alter the tissue microenvironment and influence cellular functions.^54^ Given that the og group experienced a longer monolayer expansion period, the observed differences in the functional heterogeneity outcomes of the three isolation methods could partially be affected by cellular senescence introduced by the extended monolayer culture time.

In contrast to the notable positive correlations between the identified panel of cell migration‐related genes and microtissue mechanical property for the d‐ and og‐isolated MFC, the dog‐isolated MFC expressed a panel of cell migration‐inhibitory genes, including *TNKS1BP1* and *PTPN12*, which are negative regulators of cell motility.^55^, ^56^ Interestingly, the expression of *AFAP1*, which encodes for Actin Filament‐Associated Protein 1 and of known mechanosensory functions,^57^ positively correlated with the mechanical property of microtissue pellets developed from dog‐method isolated cells (Figure 5B). For dog‐isolated MFC, the list of mechano‐cytoskeleton negatively correlated genes included genes that are involved in ciliogenesis. For example, *P1K3R4*, which has been implicated in regulating the length of primary cilia in fibroblasts^58^; and *MYO6*, which has been observed to maintain the structure of cilia in cochlear hair cells.^59^ Moreover, the list also included genes involved in cellular motility and migration: *WNK1*, which regulates cell migration and invasiveness in a variety of tumour cell types^60^: and *KIF16B*, which regulates the intracellular motility and cytoskeletal remodelling.^61^

**FIGURE 6:**
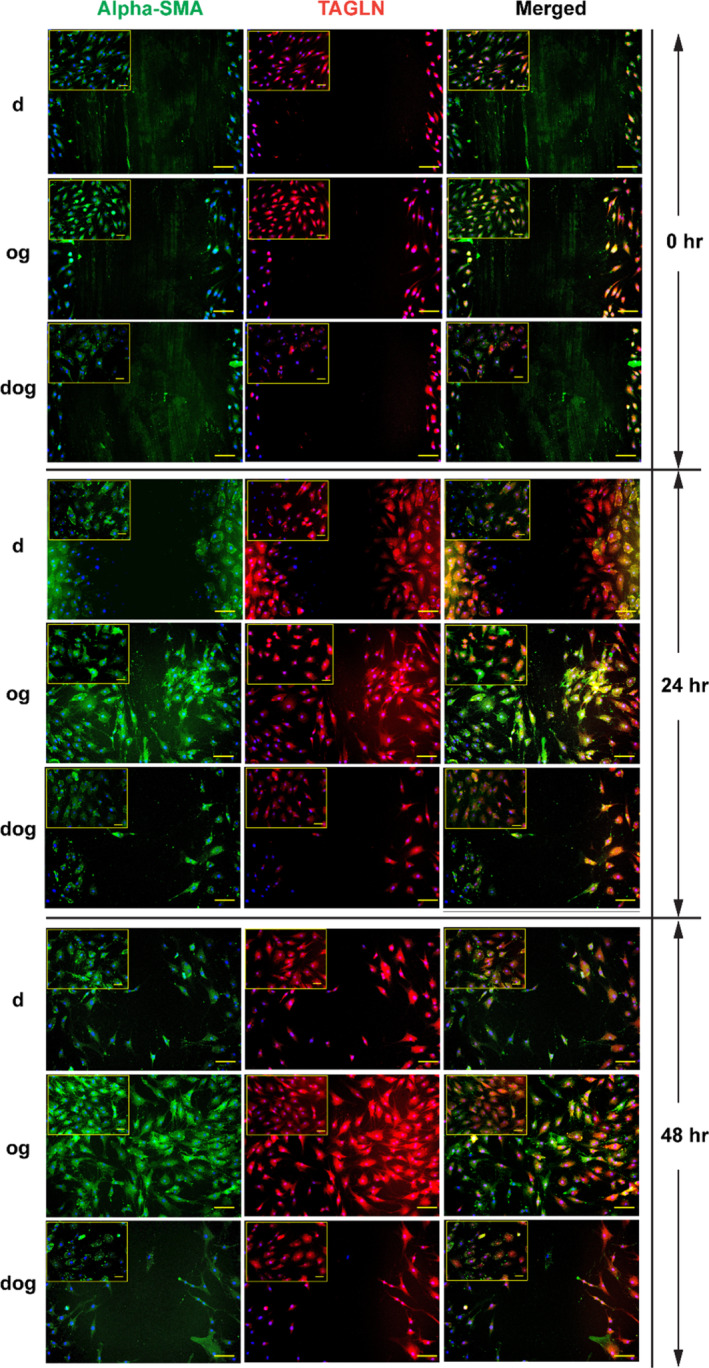
Cell Migration Capacity and Phenotypic Characteristics by Wound Scratch Assay. Wound scratch assay comparing cell migration capabilities of meniscus fibrochondrocytes (MFC) isolated via d‐, og‐ and dog methods at 0‐, 24‐ and 48‐h post‐scratch, with immunofluorescent labelling for transgelin (TAGLN) and alpha‐smooth muscle Actin (ACTA2). Scale bars: Main: 100 μm, Insert: 45 μm.

Given the notable positive relationships between the expression of genes associated with cell migration and microtissue mechanical property in the context of d‐ and og‐method isolated MFC, and the contrary regarding dog‐method isolated MFC, we decided to investigate the cell migratory capacities of MFC from the three methods in a wound scratch assay model (Figure 6). Given the context of highest expression level of *ACTA2* and *TAGLN* in the og‐derived microtissue pellets amongst the three groups, we also studied the expression levels of alpha‐smooth muscle actin (α‐SMA), the protein encoded by *ACTA2*, and transgelin, the protein encoded by *TAGLN* on the monolayer MFC in the wound scratch assay using immunofluorescence. Og‐isolated MFC showed notable migration as soon as 24 h after the scratch, compared to d‐isolated MFC, which showed intermediate cell migration, while no migration was observed in dog‐isolated MFC at this time point. At 48 h, og‐isolated cells fully populated the scratch wound area, while d‐isolated cells partially populated the scratch wound area. In contrast, after 48 h, dog‐isolated MFC barely populated the scratch wound area. Moreover, we observed that the og‐derived migratory cells exhibited intense staining for both α‐SMA and transgelin. These results aligned with the findings of Kambic et al., who demonstrated that α‐SMA positive migratory meniscus cells were responsible for the seamless repair.^62^ Moreover, high magnification images showed cell morphological differences between the differentially isolated cells (Figure 6). The d‐isolated MFC showed a mixed population of round, polygonal and fibroblast‐like cells, og‐isolated MFC were predominantly spindle‐shaped with myofibroblast‐like appearance, and dog‐isolated MFC showed predominantly round and polygonal cells (Figure 6).

Ghadially et al. were the first to identify the presence of migratory myofibroblast‐like cells in injured portions of human menisci.^63^ *ACTA2* expression is a typical characteristic of connective tissue repair; any injury to the tissue triggers inflammatory response, which in turn leads to activation of latent TGF‐β embedded in the matrix. Upon activation, it leads to release of TGF‐β1, causing fibroblasts to transform into *ACTA2* expressing contractile myofibroblasts.^64^ In our study, the activation of *ACTA2* and the expression of α‐SMA could be attributed to the stress experienced during the surgery and cell isolation process. The meniscus tissue was diced into small pieces, causing mechanical disruption and cell death adjacent to the lesion edge.^65^ This mechanical stress, combined with enzymatic digestion and culture expansion, may mimic the conditions of injury and trigger the activation of *ACTA2*. The fact that MFC can demonstrate myofibroblast‐like phenotype highlights their potential role in meniscal tissue repair and regeneration. Particularly, the og‐isolated MFC population showing predominantly myofibroblast‐like features could be used for such applications.

The observed low level but notable expression of *ACTA2* and *TAGLN* in microtissue pellet and the presence of α‐SMA protein on monolayer culture from d and dog methods could be because of the presence of TGF‐β in the chondrogenic induction medium^66^, ^67^ and multiple expansion of these cells on tissue culture plastic plate, since substrate stiffness imparts some degree of α‐SMA expression in the cultured cells, as observed and reported by Talele et al.^68^ In comparison, for og method, we observed significantly higher expression of *ACTA2* and *TAGLN* in the microtissue pellet, along with the intense expression of α‐SMA and transgelin and the exhibition of myofibroblast‐like morphology in the monolayer indicating the presence of migratory myofibroblast like cells. Furthermore, given the important role of presence of TGF‐β and stiffness of culture surface on the expression of α‐SMA, future studies with varied concentrations of TGF‐β supplement and culture substrate stiffnesses for MFC isolated with different methods are warranted to shed some more light on the mechanism of *ACTA2* expression regulation in MFC.

In conclusion, we have demonstrated that the novel dog method of meniscus cell isolation provides a source of MFC with high chondrogenic capacity. This method is particularly suited for isolating cells for the regeneration of the avascular region of the meniscus. In contrast, og‐isolated MFC exhibit high migratory capacity, making them ideal candidates for meniscus repair. Moreover, a mixed population of cells derived from both the dog and og methods can be used in a balanced manner to modulate avascular meniscus repair and regeneration. Future in‐depth studies are required to elucidate the unique features of these differentially isolated MFC populations and to translate these findings into clinical applications for meniscus repair and regeneration.

## AUTHOR CONTRIBUTIONS

Conceptualisation: ZM, SC, ABA. Methodology: ZM, SC, XL, AMS, MK, ABA. Investigation: ZM, SC, XL, ABA. Visualisation: ZM, SC, XL, EZ. Supervision: ABA. Writing—original draft: ZM, SC, EZ. Please Writing—review & editing: ZM, SC, ABA.

## FUNDING INFORMATION

Financial support was provided by the Canadian Institutes for Health Research (MOP 125921, PJT185932, PS159661), Natural Sciences and Engineering Research Council of Canada (RGPIN‐2018‐06290, RTI‐2019‐00310), Canada Foundation for Innovation (CFI 33786), Cliff Lede Family Charitable Foundation (RES00045921), University Hospital of Alberta Foundation (UHF; RES0028185; RES0045921), Faculty of Medicine & Dentistry, University of Alberta‐Precision Health Signature Area Innovator Award (Mid‐Career; 2022), Edmonton Civic Employees Charitable Assistance Fund, Wu Tsai Human Performance Alliance Agility Project Award, Women and Children's Health Research Institute's Graduate Studentship, Alberta Innovates Postdoctoral Recruitment Fellowship and Alberta Innovates Health Solutions Summer Studentship.

## CONFLICT OF INTEREST STATEMENT

The authors declare that they have no competing interests.

## IRB STATEMENT

Human inner meniscus tissues from partial meniscectomies were collected with approval from the University of Alberta Health Research Ethics Board—Biomedical Panel (Study ID: #Pro00018778). This study involved 11 males aged 14 to 39 and three females aged 16, 17 and 21 (Supplementary Table S3).

## Supporting information


**Data S1.** Supplementary Information.

**Video S1.** Video recording and animation of the stepwise relaxation of the microtissue pellets. The left panel: the video recording of the stepwise relaxation compression test. Top right panel: the animated profile of programmed displacement over time. Each step compression is 2.5% strain at the strain rate of 2% per second, followed by a 300‐s relaxation period. Bottom right panel: The animated force profile measurement in response to the applied displacement protocol.

## Data Availability

All data needed to evaluate the conclusions in the paper are present in the paper and/or the Supplementary Materials. RNA‐Seq data was deposited into the Gene Omnibus Database (GEO) under accession number GSE259265 and will be available upon the publication of this study.
