# Supplementary material for: Functional heterogeneity of meniscal fibrochondrocytes and microtissue models is dependent on modality of fibrochondrocyte isolation
Source: Cell Prolif. 2024 Oct 8;58(1):e13735. doi: 10.1111/cpr.13735 (PMC11693566; doi:10.1111/cpr.13735)
Supplement: Supplementary file 1 — Data S1. Supplementary Information. [file CPR-58-e13735-s001.docx]

**This Supplementary Material file includes:**

Materials and Methods

Figs. S1

Tables S1, S2, and S3

**Other Supplementary Materials for this manuscript include the following:**

Movies S1

**Materials and Methods**

***Cell Isolation and Recovery***

The meniscus tissue used in this study was sourced from the inner avascular region of human subjects who had undergone partial meniscectomy by a sports medicine orthopedic surgeon for acute injury, but with no history of osteoarthritis. Meniscus samples were cut into small approximately 1mm³ pieces using a scalpel and divided into 2 portions: ⅓ to be used for digestion method (d), ⅔ for outgrowth (og) and digestion after outgrowth (dog). Wet weights of meniscus tissue were recorded prior to isolation by digestion, and remnant tissue wet weighed after outgrowth.

***Digestion (d)***

Meniscus fragments to undergo digestion were transferred to 50mL tubes containing 10mL of 0.15% w/v collagenase type 2 solution (Worthington, LS004176) for every 1g of tissue, then placed on an incubator shaker (Innova 4000, New Brunswick Scientific) for 22 hours at 250 rpm and 37°C. After 22 hours the reaction of collagenase type 2 was stopped by adding the DMEM complete medium (Dulbecco’s Modified Eagle’s Medium - High Glucose (D6429, Sigma-Aldrich), supplemented with 1% Penicillin-Streptomycin-L-Glutamine (PSG) (10378, Gibco), 1% 4-(2-hydroxyethyl)-1-piperazineethanesulfonic acid (HEPES)(H4034, Sigma-Aldrich) and 10% inactivated Fetal Bovine Serum (FBS)(F1051, Sigma-Aldrich)). The resulting solution was filtered through a sterile 100µm cell strainer, centrifuged at 1500 rpm for 10 minutes at 22°C, washed with Gibco Dulbecco’s Phosphate Buffered Solution (DPBS) (14040133, ThermoFisher Scientific), resuspended in DMEM complete medium, and plated in cell culture flasks. The flasks are then placed in a humidified incubator ((HeraCell 150i, ThermoFisher Scientific) under hypoxic conditions (3% oxygen, 5% CO₂, 37°C) for 48 hours to allow for recovery. After 48 hours, the attached cells were treated with trypsin-EDTA 0.05% w/v (Cellgro, 25-052-CI) and the resulting detached cells were counted and replated at 10,000 cells/cm² in DMEM complete medium for further expansion.

***Outgrowth (og)***

Meniscus fragments to undergo outgrowth were placed in petri dishes containing 20mL DMEM complete medium per plate and incubated under humidified hypoxic conditions (3% O₂, 5% CO₂, 37°C) for 14 days to allow for cells to migrate out from the fragments. An earlier trial of 7-10 days revealed that not enough cells had migrated out of the meniscus and attached to the dish. At the end of 14-day culture, meniscus fragments were removed and the remaining adherent outgrowth cells on the plates detached using Trypsin-EDTA 0.05% for 5 minutes in a 37°C incubator. The resulting detached cells were counted and replated at 10,000 cells/cm² in DMEM complete medium for further expansion.

***Digestion after outgrowth (dog)***

In this cell isolation method, the remnant meniscus fragments from the outgrowth method above were collected at the end of 14-day culture period. The fragments were treated as per the digestion method mentioned above, using collagenase type 2 digestion for 22 hours at 37°C. The resulting cells from the digested fragments were then counted and plated in a cell culture flask under hypoxic conditions for 48 hours to allow cell recovery, then replated at 10,000 cells/cm² in DMEM complete medium for further expansion.

***Cell culture expansion***

Cells were expanded to P1 at a seeding density of 10,000 cells/cm² with DMEM complete medium. Medium was changed every 3-4 days. Cells were then expanded to P2 at the same seeding density (10,000 cells/cm²). Passaging of adherent cells was done using trypsin-EDTA 0.05%. All cell expansion experiments were incubated under humidified hypoxic conditions (3% O₂, 5% CO₂, 37°C).

***Fluorescent Activated Cell Flow Cytometry***

All primary monoclonal antibodies used herein were directly conjugated antibodies to fluorescein isothiocyanate (mAb-FITC) or to phycoerythrin (mAb-PE) (Supplementary Table 1). At the end of P2, the cells were analyzed on a BD FACS Canto II flow cytometer (Becton Dickinson) after detachment from the culture flask by 0.05% w/v trypsin-EDTA. Staining buffer was prepared with PBS containing 1% w/ v BSA (Sigma). The cells were re-suspended in 4°C cold staining buffer at 5×10^6^/ml. The cells were dispensed into sample tubes (12 × 75 mm polystyrene round-bottom tubes, Becton Dickinson) in 20 μl aliquots and incubated for 15 minutes with the antibodies at 4°C. All incubations were implemented in sample tubes at room temperature in the dark and all washing steps were performed by a combination of centrifugation (400 g, 5 minutes) and aspiration of supernatant. Staining buffer (200 μl) was then added to the tubes and the cells were incubated for 10 additional minutes. After removal of the supernatant by centrifugation, cells were washed with PBS and kept cold before analysis by flow cytometry. Non-specific staining was assessed using relevant isotype controls. Single color immunofluorescence analysis for the different surface markers was performed with mAb-FITC and mAb-PE. Data acquisition was performed with BD FACS Diva™ software (Becton Dickinson). FITC and PE emission was detected using the blue laser at 488 nm and the detection filter 525/50 and 576/26 respectively. For each sample a region for live cells was defined, according to the Forward Scatter (FSC) and Side Scatter (SSC) signals, which also exclude the aggregated cells and fragments from the analysis. Data analysis was performed with FlowJo Version 10. For each expression marker analyzed, percentage of positive cells and the level of marker expression were calculated. Based on the signal of isotype control, cells were considered positive for a surface marker when the percentage of positive cells for that surface marker was ≥ 6%. Flow cytometry was performed at the University of Alberta’s flow cytometry core of the Faculty of Medicine and Dentistry, with grant support from the Canadian Institutes of Health Research (CIHR) and financial support from the Faculty of Medicine and Dentistry. The number of donors included for FACS was 3 for all three groups.

***Microtissue pellet Formation and Tissue Culture***

At the end of P2, cells were detached using 0.05% w/v trypsin-EDTA and microtissue pellet were formed using these cells at a density of 0.25 million cells per pellet. To be specific, the detached cells were resuspended in 250µL of standard serum-free chondrogenic medium (SFMT3DAP) consisting of DMEM-High Glucose (Sigma-Aldrich), PSG (10378, Gibco), HEPES (Sigma-Aldrich), 1x insulin/transferrin/selenium (ITS+) Premix (Corning 354352), 10ng/mL TGFβ3 (ProSpec), 100nM dexamethasone, 50µg/mL ascorbic acid 2-phosphate, 125µg/mL human serum albumin, and 40µg/mL proline and transferred to a 1.5mL sterile conical microtube with screw caps (BioBasic, Canada). The cell suspension was centrifuged at 1500 rpm for 10 minutes to form 3D microtissue pellets, then placed in hypoxic conditions (3% O₂, 5% CO₂, 37°C) for 3 weeks to culture. Chondrogenic medium was changed twice weekly for the three weeks culture period.

***Physical characteristics of meniscus fibrochondrocytes (MFC) and microtissue pellets***

Meniscus fibrochondrocytes (MFC) in *in vitro* culture were observed under a Zeiss Primovert inverted light microscope fitted with a Canon EOS T6i DSLR camera 48 hours after recovery for each methods, as well as at the end of P2. Gross microtissue pellet morphology was observed for each sample after 3 weeks of culture for each isolation method. Gross morphology images were captured on a Zeiss Stemi 2000-C stereo microscope fitted with an Axiocam 208 color camera. The gross morphology images were processed by Image J software (U.S. National Institutes of Health, Bethesda, Maryland, USA) and surface area calculations were performed. Microtissue pellet wet weights were recorded at room temperature using a Mettler Toledo AB54-S analytical balance.

***Biochemical assessment for glycosaminoglycans (GAG) and DNA content***

After three weeks of culture, pellets were washed with PBS and frozen at −80°C for 4 hours, before addition of 250µL of Proteinase K solution (Sigma-Aldrich P2308) for digestion overnight at 56°C. Once fully digested, samples underwent 1,9-dimethylmethylene blue (DMMB) (Sigma Aldrich, USA, 341088) assay for detection of sulfated glycosaminoglycans (GAG) and DNA was quantified using a CyQUANT Cell Proliferation Assay (ThermoFisher Scientific C7026). The number of donors included for GAG and DNA measurement was 9 for d and og groups, and 13 for dog groups.

***Histological and immunofluorescence assessments***

Pellets for histology and immunofluorescence staining were fixed in 1mL of formalin (Fisher 305-510) overnight at 4°C. Afterwards, they were dehydrated in ethanol and embedded in paraffin wax. The pellets were then sliced to 5µm thick sections using a microtome. The sections underwent Safranin-O (Sigma-Aldrich S2255-25G) and Fast Green (FGF) (Sigma-Aldrich F7258-25G) staining for visualization of sulfated glycosaminoglycan ECM deposition.

For type II collagen immunofluorescence staining, protease XXV (Thermo Scientific AP-9006-005) and hyaluronidase (Sigma-Aldrich H6254) were applied to the sliced sections to retrieve antigen. Following the antigen retrieval, sections were blocked by 5% w/v BSA in PBS, labelled with mouse anti-human collagen type II antibody (1:200, Developmental Studies Hybridoma Bank II-II6B3-S), and incubated at 4°C overnight. On the second day, sections were washed and incubated for 30 minutes at room temperature with secondary antibody goat anti-mouse IgG H&L Alexa Fluor 488 (1:200, Thermo Fisher A32723) for type II collagen visualization, and 4’,6-diamidino-2-phenylindole (DAPI) was applied afterwards for nuclei visualization.

For immunofluorescence staining of transgelin and alpha smooth muscle actin, antigen retrieval was performed using the IHC-TekTM Epitope Retrieval Steamer Set, and sections were permeabilized with 1% w/v BSA supplemented with 0.4% v/v Triton X-100 in PBS. Processed sections were then blocked with 5% w/v BSA in PBS, labeled with mouse anti-alpha-smooth muscle actin antibody (1:200, Invitrogen MA5-11547) and rabbit anti-transgelin peptide 1 antibody (1:200, Developmental Studies Hybridoma Bank AB_2617350), followed by an overnight incubation at 4°C. On the second day, slides were washed and incubated for 45 minutes at room temperature with goat anti-mouse IgG H&L Alexa Fluor 488 (1:200, Thermo Fisher A32723) for alpha-smooth muscle actin visualization and goat anti-rabbit IgG Alexa Fluor 594 (1:200, Thermo Fisher A11012) for transgelin visualization. DAPI was used for nuclei labeling. Immunofluorescence imaging was conducted using the Nikon Eclipse Ti-S microscope.

***Scanning and transmission electron microscopy***

Microtissue pellet ultrastructure and surface collagen fiber properties were imaged using scanning electron microscopy (SEM). Microtissue pellets were processed for SEM as we have previously described(1). SEM images were captured using a Hitachi Model S-4800 scanning electron microscope (Hitachi, Japan) at the University of Alberta’s Cell Imaging Core. For the fiber size quantification, fibers were manually selected and measured using Bluebeam software (Bluebeam, Inc., USA). The selection process was random and unbiased, with an effort to measure all fibers visible in the 3000x magnification images. The measurements were used to generate a histogram illustrating the distribution of fiber diameters in the samples.

Microtissue pellets were processed for transmission electron microscopy (TEM) analysis at the University of Alberta’s Cell Imaging Core. Briefly, tissue processing was performed using an automated Leica EM TP Tissue Processor. Ultrathin sections were made by a Leica EM UC7 ultramicrotome and diamond knife. Four nm thick carbon evaporation was performed using a Leica EM ACE600 Carbon Evaporator. The ultrathin sections were imaged under a Hitachi H-7650 Transmission Electron Microscope at 80keV high tension.

***Scratch wound assay***

d, og and dog MFC from one donor were seeded at a density of 6 × 10^5^ cells/well into 6 well tissue culture plates and cultured for 48 hours with DMEM complete medium for the cells to reach 80% confluency. A cell-free line was created by scratching the centre of the cell cultured well with a sterile 200μL pipette tip as control. Before imaging, the DMEM complete medium was removed, and wells were washed with PBS to remove cellular debris. The culture plates were then reloaded with fresh DMEM complete medium. Scratched wells were stained for the same mouse anti-alpha-smooth muscle actin antibody and rabbit anti-transgelin peptide 1 antibody as used for immunofluorescence staining. Images were taken immediately (0 hours), 24 hours and 48 hours after the scratch using the ZOE Fluorescent Cell Imager (BioRad, Ontario, Canada).

***Micromechanical Characterization of Microtissue Pellets***

The mechanical properties of the microtissue pellets were evaluated by using a MicroTester G2 (CellScale Biomaterials Testing, Ontario Canada) to perform a series of stepwise stress relaxation tests (Supplement Video 1). Two technical replicates were tested for each group of each donor. The average value of these replicates was used as the equilibrium modulus for that donor for the specific group. Each pellet was placed between two platens, with the height measured as the upper platen made initial contact when descending. The stepwise testing involved 9 sequential strain steps, each consisting of a 2.5% strain ramp at a rate of 2% strain per second, followed by a relaxation period of 300 seconds at constant strain. All tested pellets reached equilibrium within the specified relaxation period, and force was recorded over time. Stress calculations were performed by normalizing the force with respect to the cross-sectional area of the pellet. The equilibrium modulus, representing the stiffness of the pellet, was defined as the slope of the strain-stress curve procured from linear regression analysis of the best-fit region. The number of donors included for the mechanical test was 6 for all three groups.

***Quantitative Real-time Gene Expression (RT-qPCR)***

After three weeks of culture, pellets of each isolation method were placed into TRIzol (Life Technologies 15596018) and frozen at -80°C. For RNA extraction, pellets were ground with Molecular Grinding Resin (G-Biosciences, 786-138PR, MO, USA) and total RNA was extracted using PuroSPIN Total RNA Purification KIT (Luna Nanotech, Canada). The RNA was reverse transcribed to complementary DNA (cDNA) using Goscript Reverse Transcriptase (A5004, Promega, WI, USA). Quantification was performed by quantitative real-time polymerase chain reaction (RT-qPCR) using gene-specific primers (Supplementary Table 2) with Takyon No Rox SYBR MasterMix dTTP Blue detection. Gene expression was normalized to geometric mean of three housekeeping genes (*β-Actin, YWHAZ, and B2M*) and the data was presented using the 2^-ΔCT^ method**.** The number of donors included for RT-qPCR measurement of *SOX9, COL2A1, ACAN, ACTA2,* and *TAGLN* was 9 for the d and og groups, and 13 for the dog groups. For *CAV1* and *CAV2*, the number of donors was 6 for all three groups. For *CNMD* and *LOX*, the number of donors was 9 for all three groups.

***Bulk Transcriptomic Profiling via Shotgun RNA Sequencing (RNAseq)***

At the end of P2, simultaneously with the setting of pellet cultures, 0.25 million cells from each donor and isolation method were removed as a monolayer culture control for RNA sequencing. These cells were immediately suspended in TRIzol (ThermoFisher 15596018) at −80°C to prevent gene expression changes. Later, RNA was extracted using the PuroSPIN Total RNA Purification KIT (Luna Nanotech, Canada) and sent to The Biomedical Research Centre at the University of British Columbia for sequencing. Bulk RNA sequencing was conducted using the Illumina NextSeq 500 platform, which generated 20 million paired end reads with a length of 42bp x 42bp. Analysis of the data was performed using Partek Flow software (Partek Inc, St. Louis, MO, USA). The raw input reads were trimmed and aligned using STAR 2.7.3a to the reference human genome hg38. Genes below 50 maximum read counts were removed, and normalization performed using Add 1.0, TMM, and Log 2.0 parameters. ANOVA was conducted for statistical analysis, with donor, biological sex, and isolation methods considered random factors. By applying p-values, adjusted p-values (q-values) and fold change (FC) criteria, differentially expressed genes (DEG) were identified. Visualization of the DEGs was mapped using volcano plots and Venn diagrams. Partek software was also used to evaluate principal component analysis (PCA), Gene Ontology (GO), and KEGG pathway enrichment analysis. The number of donors included for RNAseq was 6 for d and dog groups, and 5 for og groups.

***Statistical Analysis***

Statistical analysis for RNA sequencing data was conducted using Partek Flow software, as mentioned above. All other statistical analyses were performed using GraphPad Prism (version 10.1.2). For all quantitative measurements across the three groups, we used a matched (matching donor) one-way ANOVA with the Geisser-Greenhouse correction. Multiple comparisons were corrected using the Tukey test. The statistical significance level was set at p<0.05.

Reference:

1. Liang Y, Idrees E, Szojka ARA, Andrews SHJ, Kunze M, Mulet-Sierra A, et al. Chondrogenic differentiation of synovial fluid mesenchymal stem cells on human meniscus-derived decellularized matrix requires exogenous growth factors. Acta Biomater. 2018;80:131-43.


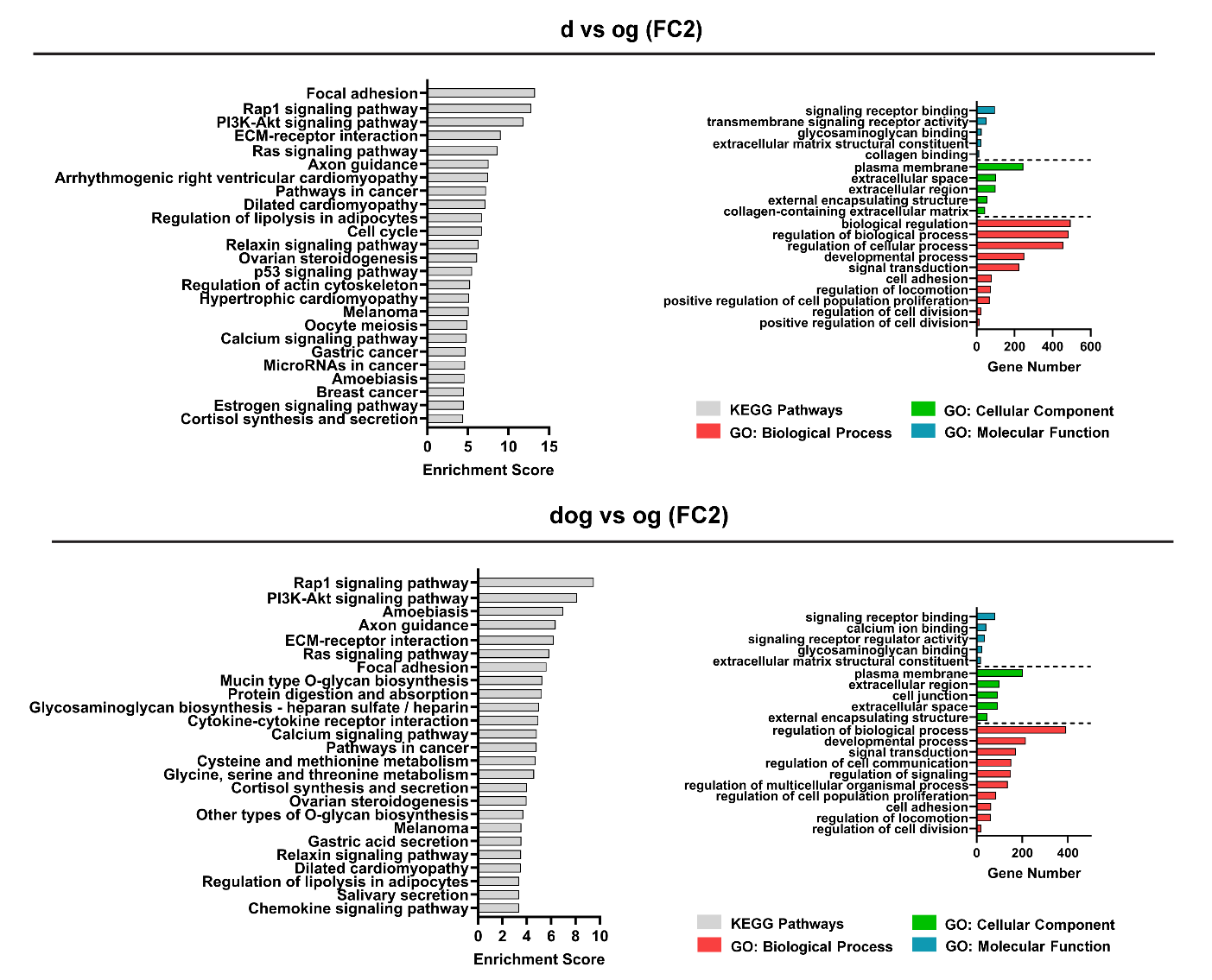


Fig. S1. Transcriptomic Analysis and Functional Enrichment of Differentially Isolated Meniscus Monolayer Cells. Functional enrichment analysis results for DEGs with a FC of 2, identifying top non-redundant Gene Ontology (GO) terms and Kyoto Encyclopedia of Genes and Genomes (KEGG) pathways.

**Table S1. List of primary antibodies used for flow cytometry.**

| Marker | Host & Isotype | Reactivity | Conjugate | Company | Lot # |
| --- | --- | --- | --- | --- | --- |
| CD34 | MsIgG1,k | Human | FITC | BD Pharmingen | 6019894 |
| CD44 | MsIgG1,k | Human | FITC | BioLegend | B162931 |
| CD73 | MsIgG1,k | Human | PE | BD Pharmingen | 3249885 |
| CD90 | MsIgG1,k | Human | PE | BD Pharmingen | 7096650 |
| CD105 | MsIgG1,k | Human | FITC | BD Pharmingen | 6119941 |
| CD146 | MsIgG1,k | Human | PE | BD Pharmingen | 6183825 |

Table S2. List of primer sequences used in RT-qPCR.

| Gene | Primer Sequences | | Accession number |
| --- | --- | --- | --- |
| Beta-Actin (*ACTB*) | Forward | AAGCCACCCCACTTCTCTCTAA | NM_001101.4 |
|  | Reverse | AATGCTATCACCTCCCCTGTGT |  |
| Beta-2-Microglobulin (*B2M*) | Forward | TGCTGTCTCCATGTTTGATGTATCT | NM_004048.3 |
|  | Reverse | TCTCTGCTCCCCACCTCTAAGT |  |
| Tyrosine 3-Monooxygenase/Tryptophan 5-Monooxygenase (*YWHAZ*) | Forward | TCTGTCTTGTCACCAACCATTCTT | NM_003406.3 |
|  | Reverse | TCATGCGGCCTTTTTCCA |  |
| Aggrecan (*ACAN*) | Forward | AGGGCGAGTGGAATGATGTT | NM_001135.3 |
|  | Reverse | GGTGGCTGTGCCCTTTTTAC |  |
| Collagen II (*COL2A1*) | Forward | CTGCAAAATAAAATCTCGGTGTTCT | NM_033150 |
|  | Reverse | GGGCATTTGACTCACACCAGT |  |
| SRY-Box 9 (*SOX9*) | Forward | CTTTGGTTTGTGTTCGTGTTTTG | NM_000346.3 |
|  | Reverse | AGAGAAAGAAAAAGGGAAAGGTAAGTTT |  |
| Alpha Smooth Muscle Actin (*ACTA2*) | Forward | TGCCTGATGGGCAAGTGA | NM_001141945.2 |
|  | Reverse | CTGGGCAGCGGAAACG |  |
| Transgelin (*TAGLN*) | Forward | GGCATGAGCCGCGAAGT | NM_001001522.2 |
|  | Reverse | TCCTCCAGCTCCTCGTCATACT |  |
| Caveolin 1 (*CAV1*) | Forward | AGGCCAGCTTCACCACCTT | NM_001753.5 |
|  | Reverse | GCAGACAGCAAGCGGTAAAAC |  |
| Caveolin 2 (*CAV2*) | Forward | ACCGGCTCAACTCGCATCT | NM_001233.5 |
|  | Reverse | CCGGCTCTGCGATCACAT |  |
| Lysyl Oxidase (*LOX*) | Forward | AGGCCACAAAGCAAGTTTCTG | NM_002317.7 |
|  | Reverse | AAATCGCCTGTGGTAGCCATA |  |
| Chondromodulin (*CNMD*) | Forward | GCGCAAGTGAAGGCTCGTAT | NM_007015.3 |
|  |  |  |  |
|  | Reverse | GTTTGGAGGAGATGCTCTGTTTG |  |

| Donor | Sex | Age |
| --- | --- | --- |
| 1 | M | 25 |
| 2 | F | 21 |
| 3 | F | 16 |
| 4 | M | 33 |
| 5 | M | 21 |
| 6 | M | 39 |
| 7 | M | 17 |
| 8 | M | 39 |
| 9 | M | 39 |
| 10 | F | 17 |
| 11 | M | 14 |
| 12 | M | 17 |
| 13 | M | 22 |
| 14 | M | 28 |

Table S3. Detailed donor information.
